# Supplementary material for: CircAnks1a in the spinal cord regulates hypersensitivity in a rodent model of neuropathic pain
Source: Nat Commun. 2019 Sep 11;10:4119. doi: 10.1038/s41467-019-12049-0 (PMC6739334; doi:10.1038/s41467-019-12049-0)
Supplement: Supplementary file 1 — Supplementary Information [file 41467_2019_12049_MOESM1_ESM.pdf]

Supplementary Information

CircAnks1a in the spinal cord regulates hypersensitivity in a rodent model of neuropathic pain

Su-Bo Zhang[1,2, a], Su-Yan Lin[2, 3, a], Meng Liu[2, 3, a], Cui-Cui Liu[1, a], Huan-Huan Ding[2, 3], Yang Sun[4], Chao Ma[1, *], Rui-Xian Guo[2], You-You Lv[3], Shao-Ling Wu[1], Ting Xu[2, *], Wen-Jun Xin[2, 3, 5, *]

[1]Guangdong Provincial Key Laboratory of Malignant Tumor Epigenetics and Gene Regulation, Department of Rehabilitation Medicine, Sun Yat-Sen Memorial Hospital, Sun Yat-Sen University, Guangzhou, 510120, China

[2]Guangdong Province Key Laboratory of Brain Function and Disease, Department of Physiology, Zhongshan School of Medicine, Sun Yat-Sen University, Guangzhou, 510080, China

[3]Neuroscience Program, Zhongshan School of Medicine, the Fifth Affiliated Hospital of Sun Yat-Sen University, Sun Yat-Sen University, China

[4]Department of Rehabilitation Medicine, the Second Affiliated Hospital of Xi'an Medical University, Xi'an, 710038, China

[5]Center for Brian Science and Brain-Inspired Intelligence, Guangdong-Hong Kong-Macao Greater Bay Areaina

[a] These authors contributed equally to this work

[*]Joint corresponding author

Supplementary Figure 1

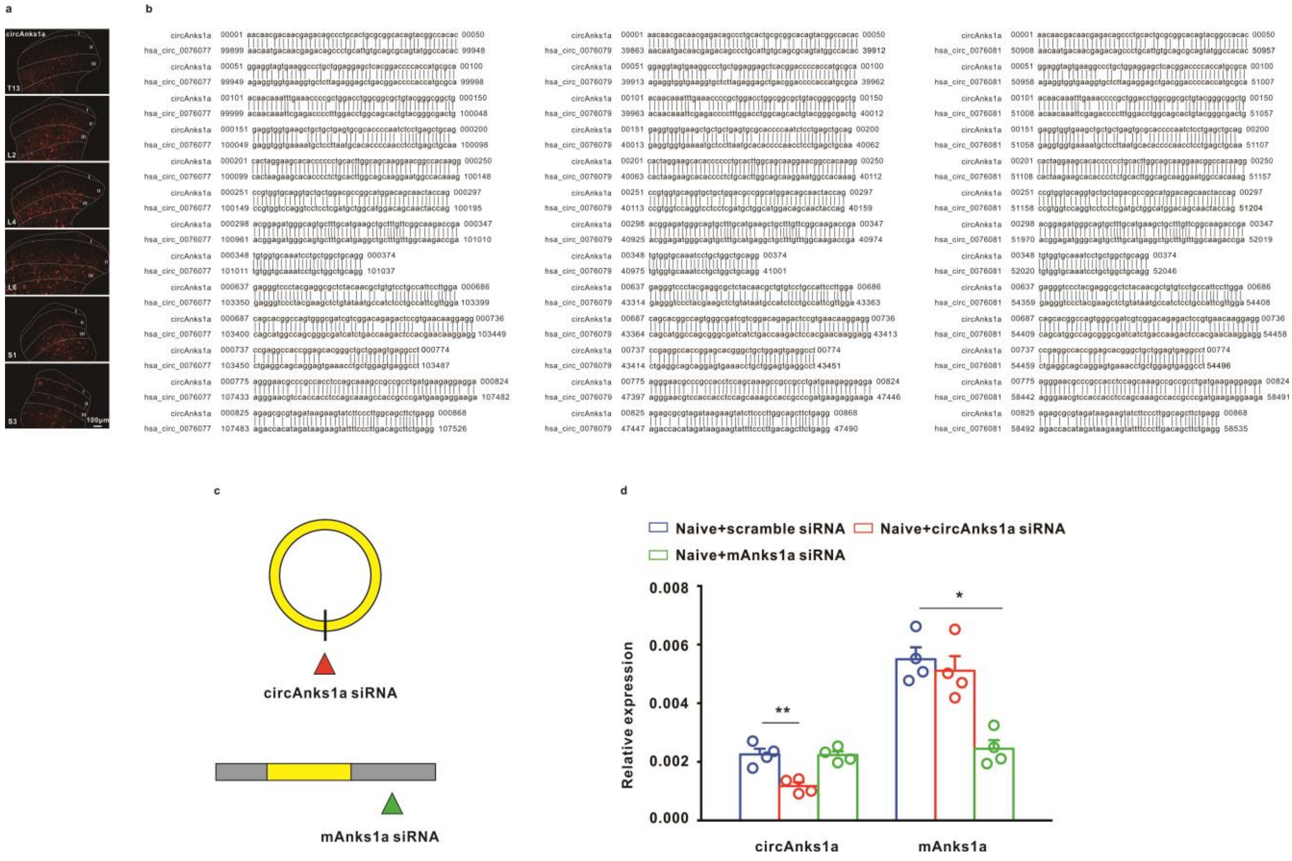

Supplementary Figure 1 (a) Expression of circAnks1a in different level of spinal from T13 to S3. (b) Full-length sequence of circAnks1a was blast with hsa_circ_0076077, hsa_circ_0076079 and hsa_circ_0076081. (c) The diagram of designed siRNA. (d) Application of circAnks1a siRNA, but not mAnks1a siRNA, significantly decreased the expression of circAnks1a in naïve animals. (n=4 in each group, *P<0.05, **P<0.01 vs the correspondence naïve group, two-tailed one-way ANOVA). Data are presented as mean ± s.e.m.

Supplementary Figure 2

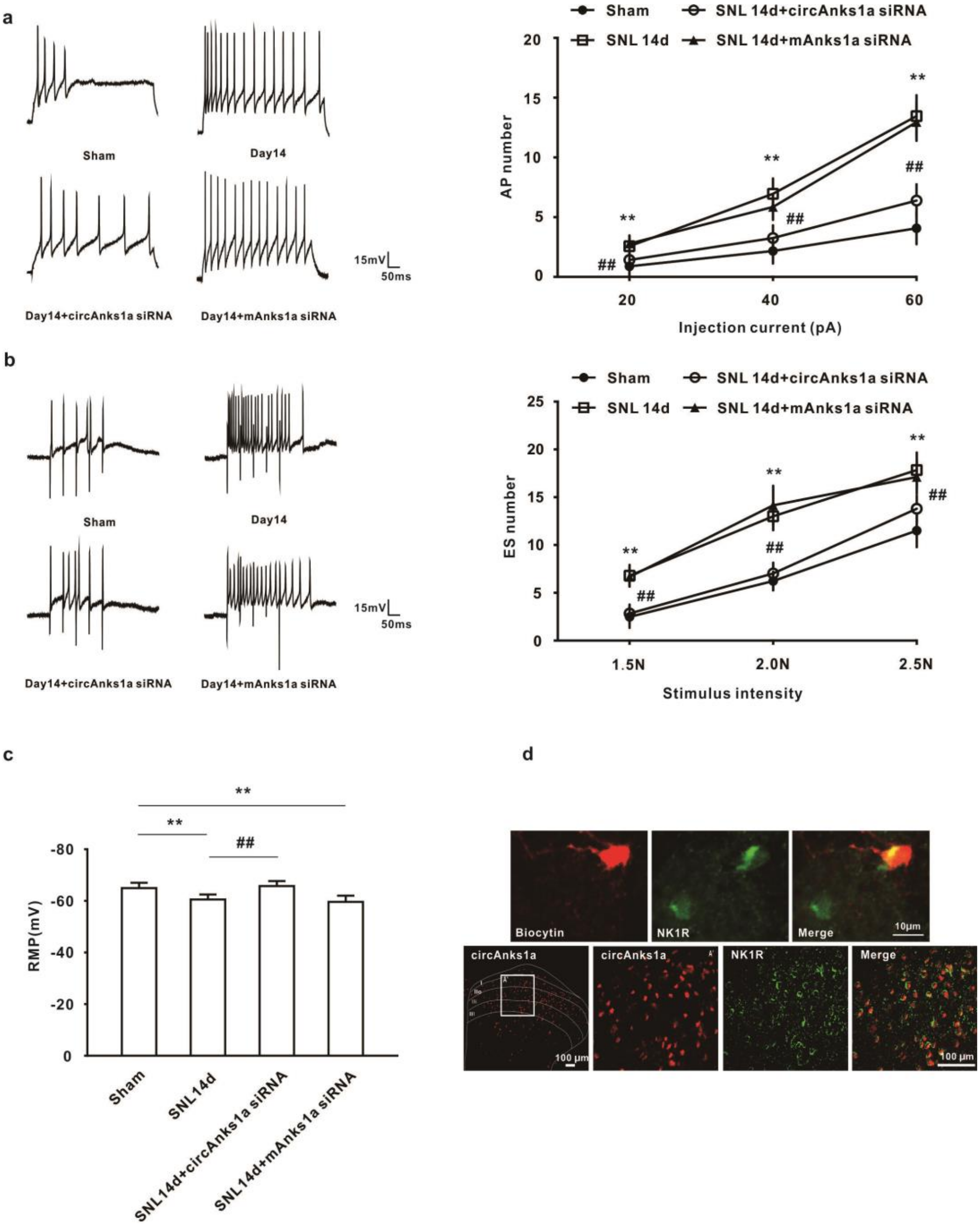

Supplementary Figure 2 The number of depolarizing current injection-induced action potentials (a), and EPSP-spike coupling (b) were increased in dorsal horn neuron at day 14 following SNL, which were alleviated by intrathecal injection of circAnks1a siRNA, but not that of mAnks1a siRNA (n = 28 for a and 25 for b, ***P* < 0.01 vs the correspondence sham group, ##*P* < 0.01 vs the SNL group, two-tailed permutation tests). (c) The positive shift of resting membrane potential were also reversed by the circAnks1a siRNA (n=28, ***P* < 0.01 vs the correspondence sham group, ##*P* < 0.01 vs the SNL group, two-tailed one-way ANOVA). (d) The electrophysiology studies were performed on NK1R-positive neurons in the spinal cord slices and circAnks1a was colocalized with the NK1R-positive cells. Data are presented as mean ±s.e.m.

Supplementary Figure 3

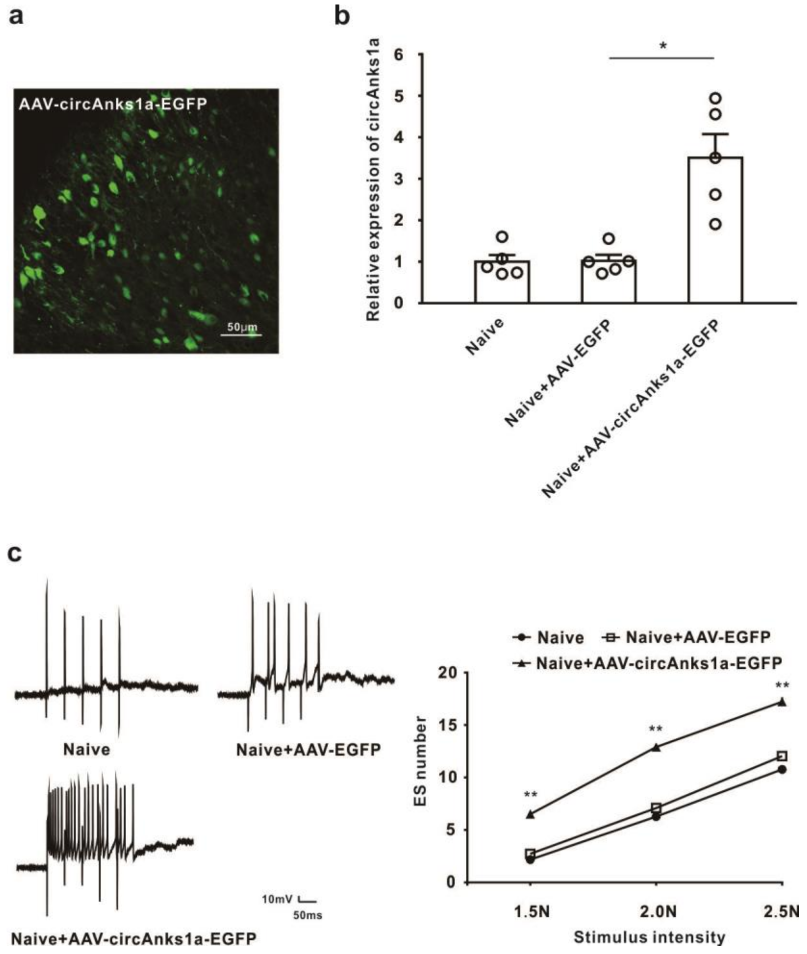

Supplementary Figure 3 Green Fluorescences (a) and increased circAnks1a level (b) in dorsal horn suggested a high transfection of AAV-circAnks1a-EGFP (n=5, *P < 0.05 vs the correspondence AAV-EGFP group, two-tailed one-way ANOVA). (c) Intraspinal injection of the recombinant AAV-circAnks1a-EGFP increased the number of EPSP-spike coupling (n = 22, **P < 0.01 vs the correspondence AAV-EGFP group, two-tailed permutation tests). Data are presented as mean ± s.e.m.

Supplementary Figure 4

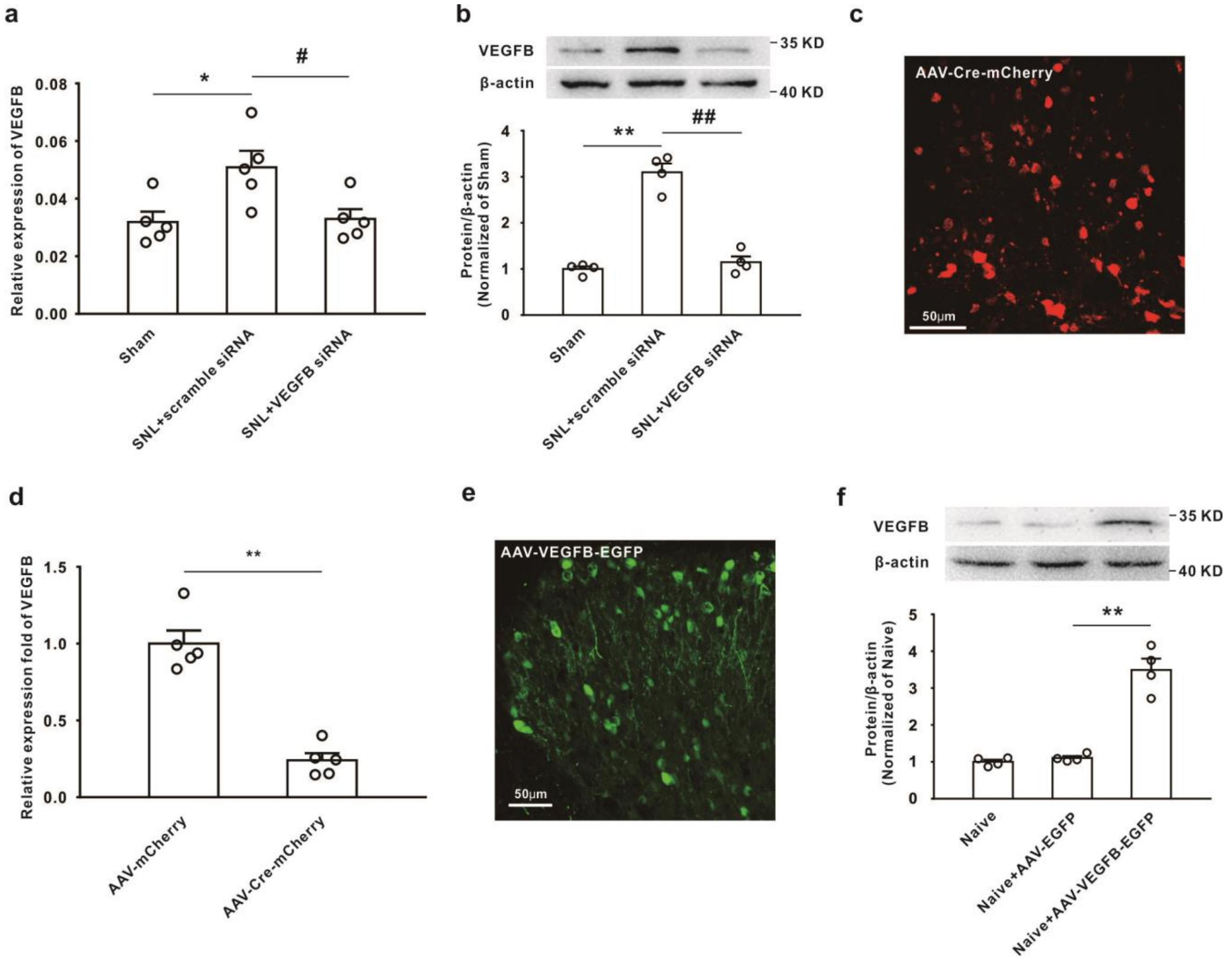

Supplementary Figure 4 Intrathecal injection of VEGFB siRNA significantly decreased the VEGFB mRNA (a) and protein (b) expression (**P* < 0.05, ***P* < 0.01 vs the sham group, #*P* < 0.05, ##*P* < 0.01 vs the scramble group, n=5 for a and n=4 for b, two-tailed one-way ANOVA). Twenty-one days after injection of AAV-Cre-mCherry, a marked red fluorescence (c) and decreased expression of VEGFB mRNA (d) were observed (***P* < 0.01 vs the AAV-mCherry group, n=3 for c and n=5 for d, two-tailed two-sample *t*-tests). A marked green fluorescence (e) and increased VEGFB protein (f) levels were observed 21 days after injection of AAV-VEGFB-EGFP (***P* < 0.01 vs the AAV-EGFP group, n=3 for e and n=4 for f, two-tailed one-way ANOVA). Data are presented as mean ± s.e.m.

Supplementary Figure 5

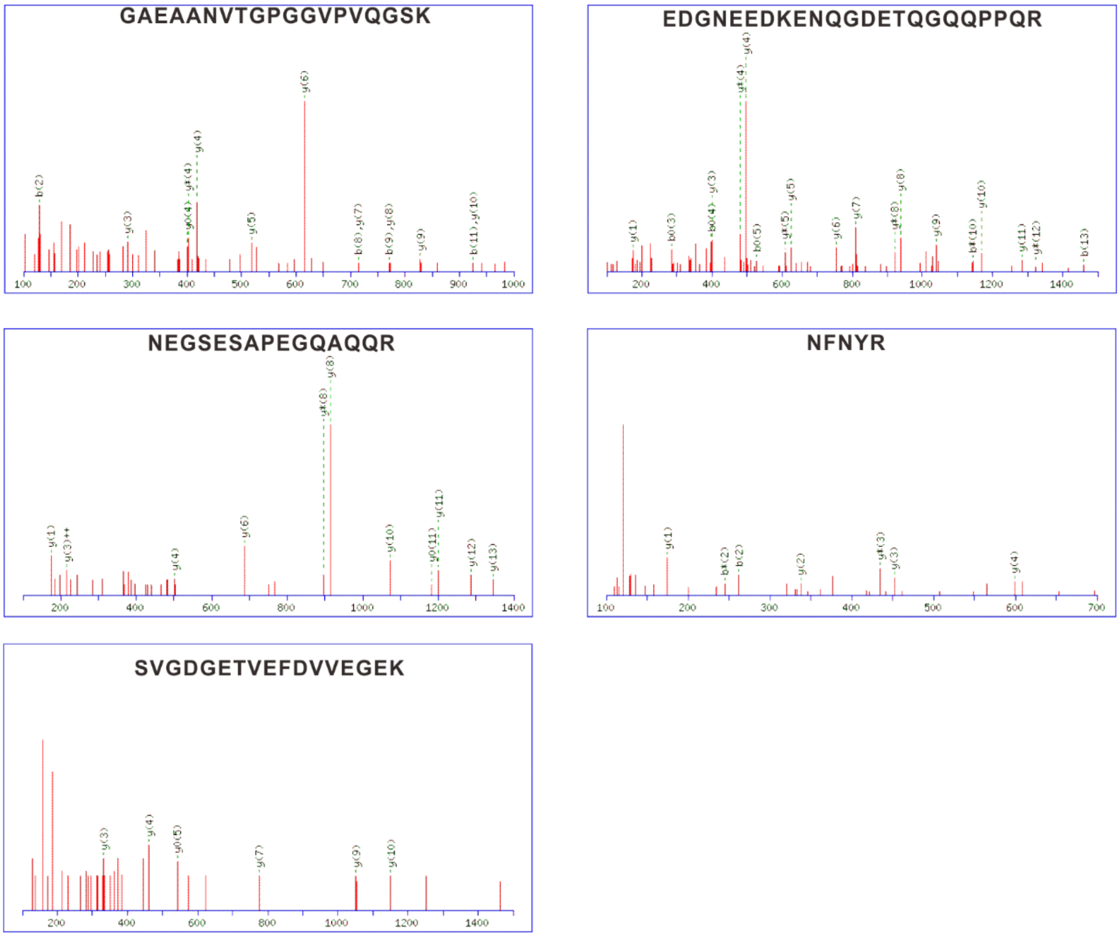

Supplementary Figure 5 The proteins pulled down by linearized circAnks1a were subjected to liquid chromatography-mass spectrometry analysis, by which five peptide sequences of YBX1 were identified.

Supplementary Figure 6

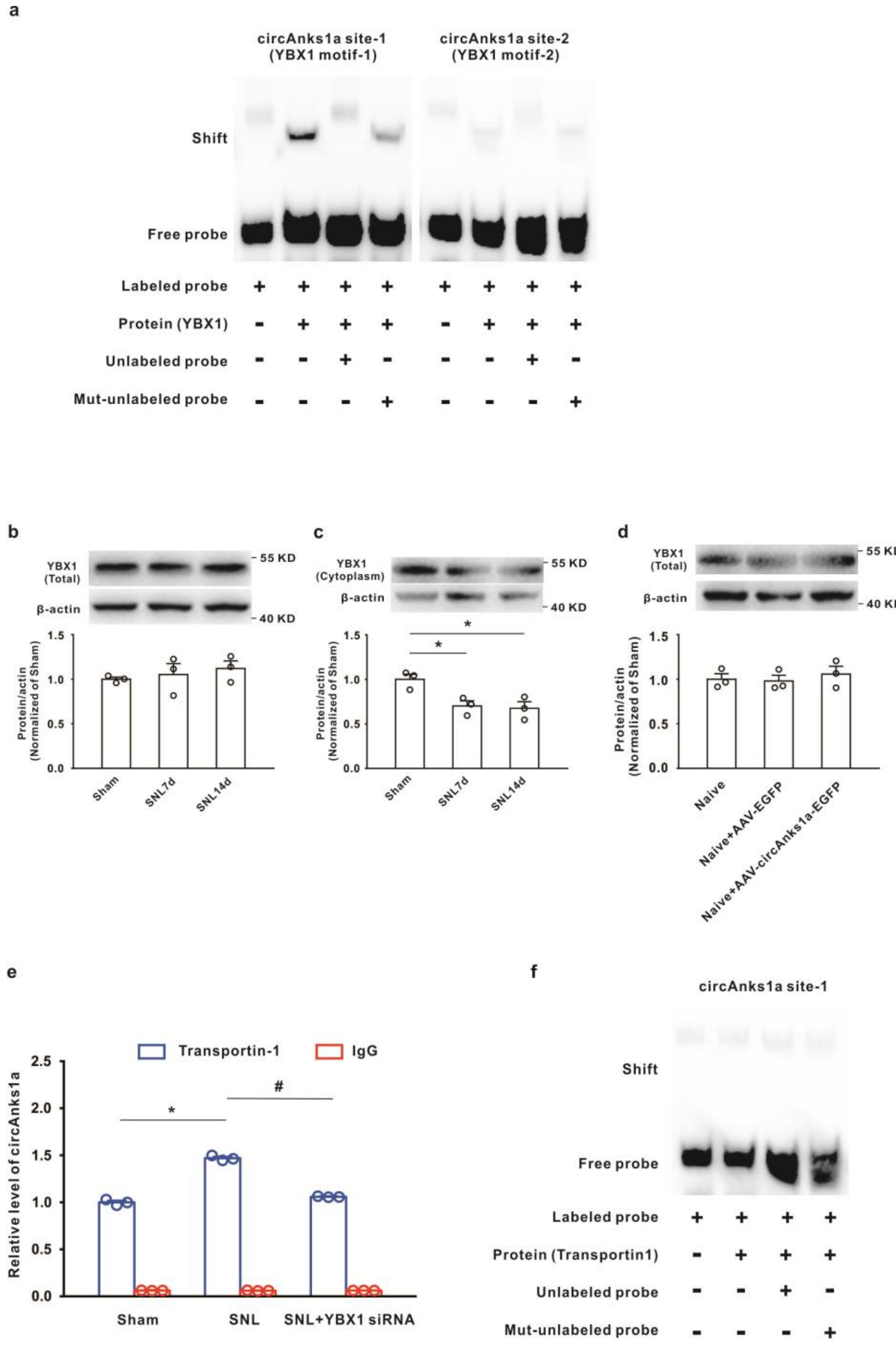

Supplementary Figure 6 (a) The EMSA experiment showed that recombination YBX1 protein can obviously bind to the circAnks1a site-1 (YBX1 motif-1), but not site-2 (YBX1 motif-2). SNL did not affect the total amount of YBX1 (b), but decreased the cytoplasmic YBX1 level (c) (*$P < 0.05$ vs the sham group, n=3, two-tailed one-way ANOVA). (d) CircAnks1a has no effect on YBX1 expression (n=3). (e) SNL significantly increased the level of circAnks1a immunoprecipiated by transporting-1 antibody by using RIP methods, which was suppressed by YBX1 siRNA (*$P < 0.05$ vs the sham group, #$P < 0.05$ vs the SNL group, n=3, two-tailed one-way ANOVA). (f) Further EMSA showed that recombination transportin-1 protein cannot bind to the circAnks1a site 1. Data are presented as mean ± s.e.m.

Supplementary Figure 7

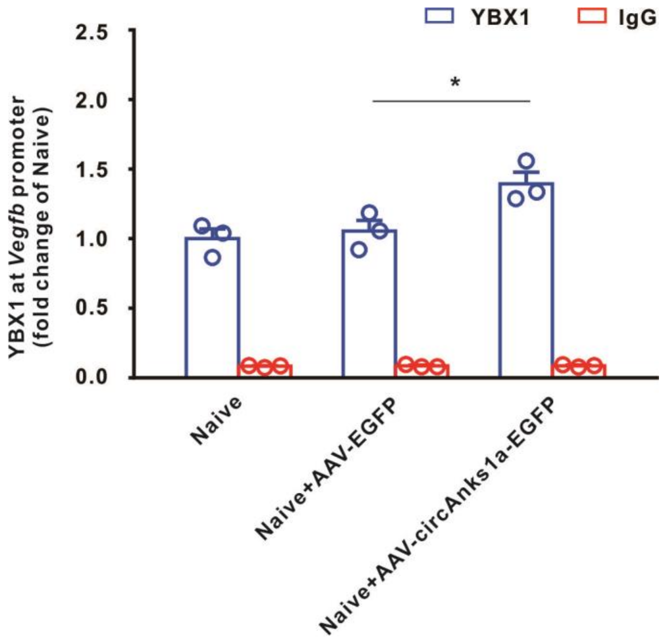

Supplementary Figure 7 The recruitment of YBX1 on the *Vegfb* promoter was significantly increased in naïve on day 21 following injection of AAV2/9-circAnks1a-EGFP. (*$P < 0.05$ vs the AAV-EGFP group, n=3, two-tailed one-way ANOVA). Data are presented as mean ± s.e.m.

Supplementary Table 1

Summary of RNA-seq datasets from the tissue of different time points (Day 7 and Day 14) and sham (Day 14)

Sample	Sham(Day14)	SNL(Day7)	SNL(Day14)
Effective clean reads	75,025,228	93,808,070	80,212,334
Mapped reads(unfusion)	65,896,226	78,607,412	68,742,172
Unmapped reads(unfusion)	9,129,002	15,200,658	11,470,162
Mapped reads(fusion)	4,224,319	6,183,831	4,855,195
Total mapped reads	70,120,545	84,791,243	73,597,367

Supplementary Table 2

Length analysis of circRNAs

Item	circRNA	Min.length	Max.length	Median length
Number	12849	57	99915	738

Supplementary Table 3

Relative expression of 21 circRNAs

Name	Relative expression of circRNAs		
	**Sham (n=4)**	**Day 7 (n=4)**	**Day 14 (n=4)**
rat_circ:chr10:4140144-4172981	1.00±0.13	0.81±0.05	0.94±0.08
rat_circ:chr3:8747385-8750638	1.00±0.12	0.69±0.12	0.79±0.06
rat_circ:chr1:16514097-16530121	1.00±0.09	0.75±0.10	0.99±0.11
rat_circ:chr10:11669353-11670969	1.00±0.08	0.72±0.12	0.74±0.04
rat_circ:chr2:172320327-172320627	1.00±0.10	0.99±0.06	1.41±0.07
rat_circ:chr9:100507197-100507524	1.00±0.04	0.85±0.08	0.87±0.07
rat_circ:chr1:250833012-250919167	1.00±0.10	0.95±0.06	1.06±0.05
rat_circ:chr14:81667646-81672500	1.00±0.13	0.91±0.18	0.85±0.09
rat_circ:chr20:7561057-7573740	1.00±0.10	1.65±0.15	2.22±0.17
rat_circ:chr6:23270238-23279721	1.00±0.10	0.82±0.13	0.85±0.08
rat_circ:chr17:23684383-23701761	1.00±0.08	1.21±0.21	1.08±0.02
rat_circ:chr8:93948882-94008016	1.00±0.04	1.34±0.14	1.38±0.18
rat_circ:chr12:19311511-19312478	1.00±0.07	0.90±0.05	1.01±0.10
rat_circ:chr10:109122252-109148654	1.00±0.14	0.82±0.09	0.84±0.09
rat_circ:chr2:188886906-188931941	1.00±0.03	0.93±0.06	1.06±0.10
rat_circ:chr18:27956412-27984766	1.00±0.08	0.94±0.10	1.11±0.09
rat_circ:chr4:84682374-84706882	1.00±0.13	0.83±0.16	0.80±0.07
rat_circ:chr3:122130941-122141403	1.00±0.03	0.98±0.10	1.13±0.18
rat_circ:chr2:172312783-172350004	1.00±0.17	0.68±0.08	0.99±0.08
rat_circ:chr1:79848478-79856028	1.00±0.11	0.72±0.05	0.88±0.16
rat_circ:chr7:11216020-11216322	1.00±0.13	1.07±0.09	1.37±0.18

Supplementary Table 4

circName	miRNA	Total Score	Total Energy	Positions
circAnks1a	rno-miR-15b-5p	467	-64.53	245 912 149
circAnks1a	rno-miR-322-5p	460	-64.63	912 149 244
circAnks1a	rno-miR-16-5p	455	-55.14	913 249 149
circAnks1a	rno-miR-494-5p	454	-62.77	380 2 981
circAnks1a	rno-miR-497-5p	446	-66.47	245 910 150
circAnks1a	rno-miR-324-3p	440	-65.03	872 294 577
circAnks1a	rno-miR-15a-5p	440	-47.62	912 252 152
circAnks1a	rno-miR-103-1-5p	436	-59.51	929 192 303
circAnks1a	rno-miR-195-5p	428	-50.56	249 912 151
circAnks1a	rno-miR-770-5p	318	-54.45	238 333
circAnks1a	rno-miR-1843b-5p	313	-46.77	501 764
circAnks1a	rno-miR-22-3p	311	-42.33	831 214
circAnks1a	rno-miR-344b-5p	300	-41.21	391 784
circAnks1a	rno-miR-320-5p	297	-48.06	890 572
circAnks1a	rno-miR-330-3p	295	-38.79	297 313
circAnks1a	rno-miR-152-3p	292	-29.06	10 989
circAnks1a	rno-miR-761	292	-42.42	52 346
circAnks1a	rno-miR-3548	292	-40.66	295 874
circAnks1a	rno-miR-107-5p	288	-34.29	929 192
circAnks1a	rno-miR-344g	288	-48.35	390 849
circAnks1a	rno-miR-666-3p	286	-40.63	180 148
circAnks1a	rno-miR-449c-5p	286	-40.19	12 991
circAnks1a	rno-miR-148b-3p	280	-24.88	9 988
circAnks1a	rno-miR-214-3p	280	-31.33	53 347

circAnks1a	rno-miR-148a-3p	280	-23.42	10 989
circAnks1a	rno-miR-496-5p	168	-22.5	382
circAnks1a	rno-let-7b-5p	167	-27.05	517
circAnks1a	rno-let-7a-5p	163	-21.52	517
circAnks1a	rno-miR-410-5p	163	-20.11	381
circAnks1a	rno-miR-3558-3p	162	-21.56	508
circAnks1a	rno-miR-292-3p	160	-33.08	116
circAnks1a	rno-let-7c-5p	159	-23.5	517
circAnks1a	rno-miR-331-5p	156	-20.22	501
circAnks1a	rno-let-7i-5p	156	-19.48	517
circAnks1a	rno-miR-293-3p	156	-22.43	19
circAnks1a	rno-let-7d-5p	155	-21.76	517
circAnks1a	rno-miR-339-3p	155	-27	636
circAnks1a	rno-let-7e-5p	155	-20.83	517
circAnks1a	rno-miR-6334	155	-36.49	843
circAnks1a	rno-miR-3574	154	-26.35	129
circAnks1a	rno-miR-935	152	-19.72	580
circAnks1a	rno-miR-1843a-5p	152	-24.55	506
circAnks1a	rno-miR-615	151	-23.68	71
circAnks1a	rno-miR-24-3p	149	-18.77	584
circAnks1a	rno-miR-92a-2-5p	149	-19.34	74
circAnks1a	rno-miR-291a-5p	149	-24.79	853
circAnks1a	rno-miR-760-5p	149	-19.17	838
circAnks1a	rno-miR-486	149	-25.1	124
circAnks1a	rno-miR-3065-3p	148	-20.9	242
circAnks1a	rno-miR-103-3p	147	-26.73	245

circAnks1a	rno-miR-107-3p	147	-24.61	245
circAnks1a	rno-miR-351-3p	146	-19.45	596
circAnks1a	rno-miR-25-3p	146	-20.91	339
circAnks1a	rno-miR-500-5p	146	-21.78	218
circAnks1a	rno-miR-343	145	-27.55	40
circAnks1a	rno-miR-6331	145	-30.7	726
circAnks1a	rno-miR-15a-3p	145	-24.72	27
circAnks1a	rno-miR-193a-3p	144	-18.76	680
circAnks1a	rno-miR-204-3p	144	-23.86	424
circAnks1a	rno-miR-3571	144	-18.88	484
circAnks1a	rno-miR-1249	144	-22.8	801
circAnks1a	rno-miR-1306-5p	144	-28.24	135
circAnks1a	rno-let-7a-2-3p	143	-24	121
circAnks1a	rno-miR-125b-2-3p	143	-26.38	849
circAnks1a	rno-miR-204-5p	143	-19.35	749
circAnks1a	rno-miR-205	143	-23.94	41
circAnks1a	rno-miR-211-5p	143	-18.45	749
circAnks1a	rno-miR-222-5p	143	-19.3	585
circAnks1a	rno-miR-329-5p	142	-17.85	499
circAnks1a	rno-miR-193a-5p	142	-19.45	326
circAnks1a	rno-miR-665	142	-18.64	173
circAnks1a	rno-miR-6314	142	-21.35	874
circAnks1a	rno-miR-30b-3p	141	-26.52	510
circAnks1a	rno-miR-135a-3p	141	-17.13	628
circAnks1a	rno-miR-503-5p	141	-22.18	149
circAnks1a	rno-miR-196c-5p	141	-18.38	519

circAnks1a	rno-miR-328a-5p	140	-23.24	880
circAnks1a	rno-miR-34a-5p	140	-18.3	406
circAnks1a	rno-miR-300-3p	140	-22.4	300
circAnks1a	rno-miR-874-3p	140	-22.49	869
circAnks1a	rno-miR-667-5p	140	-21.58	672
circAnks1a	rno-miR-711	140	-21.35	624
circAnks1a	rno-miR-3593-5p	140	-22.34	718
circAnks1a	rno-miR-193b-3p	140	-17.19	682

Supplementary Table 5

Specific primer sequences

Gene	Primer	Sequence
circAnks1a (rat)	Forward	5'-GGAAGAGCGCGTAGATAAGAAGTA-3'
	Reverse	5'-AGGGCTGTCTCGTTGTCGTT-3'
Anks1a (rat)	Forward	5'-CTCCATCGACACCGTGAAGAA-3'
	Reverse	5'-GAGGCGATAATGCGTTTGC-3'
Vegfb(rat)	Forward	5'-ATGGGTAATGTGGTCAAACAACTC-3'
	Reverse	5'-CTCGGGTACTGGATCATGAGG-3'
Vegfb(mouse)	Forward	5'-CATGGATAGACGTTTATGCACG-3'
	Reverse	5'-ACTAGTTGTTTGACCACATTGC-3'
β-actin (rat)	Forward	5'-GGAGATTACTGCCCTGGCTCCTA-3'
	Reverse	5'-GACTCATCGTACTCCTGCTTGCTG-3'
rat_circ: chr10:4140144-4172981	Forward	5'-GTGGATGCCTTGAGGAGGAAG-3'
	Reverse	5'-GAGGATGTCAGTGTCAGCATC-3'
rat_circ: chr3:8747385-8750638	Forward	5'-GTTCAGAGAAGGCGTCGTGGT-3'
	Reverse	5'-GCTGCTTCCATTTTCGCCATTC-3'
rat_circ: chr1:16514097-16530121	Forward	5'-CAGAGGTGATACGATCCTGTG-3'
	Reverse	5'-CGCTTCAACATTTCAGCCTCCT-3'
rat_circ: chr10:11669353-11670969	Forward	5'-GCCAAGTTGCCCATTGTGCAT-3'
	Reverse	5'-TGTGCGGGTACAATTCCCACT-3'
rat_circ: chr2:172320327-172320627	Forward	5'-TGGGTGAGAGTGGGATTGTG-3'
	Reverse	5'-AACGGTATCAGCTGCTGTTT-3'
rat_circ: chr9:100507197-100507524	Forward	5'-GCATTGGGTGTCAGGGGTTAG-3'
	Reverse	5'-GGGATGGAATGAGACCTGGTG-3'
rat_circ: chr1:250833012-250919167	Forward	5'-GATGCACACTACCATGTCTAT-3'
	Reverse	5'-TGTAGCTTTCCCTTCCGAGTG-3'

rat_circ: chr14:81667646-81672500	Forward	5’-CTTGTGGAAACCGTTGGAGATG-3’
	Reverse	5’-TTGGAGCCAACTAGATGTTCAG-3’
rat_circ: chr6:23270238-23279721	Forward	5’-GCCACGCTTCCAAACTGTGAC-3’
	Reverse	5’-TCATTCACGTTGCAGCCTCTC-3’
rat_circ: chr17:23684383-23701761	Forward	5’-CAGGCAGACGGATGAGGAACG-3’
	Reverse	5’-TGTGTAGGATGCCAGGGAGAG-3’
rat_circ: chr8:93948882-94008016	Forward	5’-CACAGCGAGGGAACTTCACTG-3’
	Reverse	5’-TGGAGGGCAGGGTTAAAGGGT-3’
rat_circ: chr12:19311511-19312478	Forward	5’-CGATTGATGATGGAGCAGCGG-3’
	Reverse	5’-GGTCCCATACTTGAACTGCTTC-3’
rat_circ: chr10:109122252-109148654	Forward	5’-GCCCTGGACAAGTGTCAGGCT-3’
	Reverse	5’-GACACCTGCCAGTGCTTTCTC-3’
rat_circ: chr2:188886906-188931941	Forward	5’-CGATTCGTCATGAAGACGCTC-3’
	Reverse	5’-GCGATGATCAAACCAAGCAGG-3’
rat_circ: chr18:27956412-27984766	Forward	5’-CTGCCCTGATGGCCGACTCAT-3’
	Reverse	5’-TGTTTACCAGGGTTGTCACCTGT-3’
rat_circ: chr4:84682374-84706882	Forward	5’-ATGGAAGAGATTCTGAGCAGC-3’
	Reverse	5’-GGCAGGTCTGCTTATCACTAT-3’
rat_circ: chr3:122130941-122141403	Forward	5’-GTCTTCATCGGTGTGGGTGTG-3’
	Reverse	5’-CACTGATTTGTCAGCCTGAGTC-3’
rat_circ: chr2:172312783-172350004	Forward	5’-CCACCAGAGGACAAGCTAGAAT-3’
	Reverse	5’-GAGGTTCATCCCACTTTGTAGT-3’
rat_circ: chr1:79848478-79856028	Forward	5’-CAAGCCTGAGGTGGTCAACAT-3’
	Reverse	5’-TGGACTTAGATAGCTTGATGGTG-3’
rat_circ: chr7:11216020-11216322	Forward	5’-CAAGCTGTGCGCTGGAAGTTC-3’
	Reverse	5’-GCAAAGGTGCTATAGGGTGAG-3’
ChIP for *Vegfb* promoter	Forward	5’-GGGTGGACATCATCAGGAGAG-3’
	Reverse	5’-GACGACTTTGAAGGACTGGCT-3’

ChIRP for *Vegfb* promoter region 1	Forward	5'-CCTTCAAAGTCGTCAGGGAG-3'
	Reverse	5'-GTAAGGACAGCAGGGCTCG-3'
ChIRP for *Vegfb* promoter region 2	Forward	5'-GTCAAGGAAGCAGCAGCA-3'
	Reverse	5'-CACACCACTCTTGACCCAC-3'

Supplementary Table 6

The target sequences of siRNA

Gene	Name	Sequence
circAnks1a	siRNA	5’-GAGGACTGAAGAACAACGA-3’
Anks1a	siRNA	5’-CTAGATCAGTTATGTTCAACC-3’
Vegfb	siRNA	5’-GCAGATCATCAGAAACTTA-3’
YBX1	siRNA	5’-CCAAGGAAGACGTATTTGT-3’
transportin-1	siRNA	5’-GCATAGAGATGCAGCCTTA-3’

Supplementary Table 7

The nucleotide sequences of probes used in RNA pull-down assays (Table 7a)

Probe Name	Sequence
circAnks1a probe	5'-CTCGTTGTCGTTGTTCTTCAGTCCTCCCGTC-3'
circAnks1a random probe	5'-ATTCCTCGTCTCTGTGCGGTTCTCCGTTCCT-3'
miR-324-3p probe	5'-CCACUGCCCCAGGUGCUGCUGG-3'
miRNA random probe	5'-UUGUACUACACAAAAGUACUG-3'

The nucleotide sequences of probes used in ChIRP (Table 7b)

Probe Name	Sequence
circAnks1a-1	5'-GCGGGGTTTCAAATTTGTTG-3'
circAnks1a-2	5'-TGCTGCAGCTCAGGAGATTG-3'
circAnks1a-3	5'-AGGTCTCGGACAGTGTCAAG-3'
circAnks1a-4	5'-GAGGTCTTGTCTACATCTTT-3'
circAnks1a-5	5'-GGATGTCAGAATCAAGAGTC-3'
circAnks1a-6	5'-TGTGAGCAGCAGCTCATATG-3'
circAnks1a-7	5'-GTTGTCGTTGTTCTTCAGTCCTCC-3'
mAnks1a-1	5'-ATCTACGCAGTTCACATTCG-3'
mAnks1a-2	5'-CTTTCGAGTCAGCAACGTTG-3'
mAnks1a-3	5'-TGTTCGTTGACTCTGGTGTG-3'
mAnks1a-4	5'-GTTCTGGCTGGGGACAGTAA-3'
mAnks1a-5	5'-TGGAGGAGACCTGAGAACTG-3'

Supplementary Table 8

The nucleotide sequences of probes used in EMSA (Table 8a)

**Probe Name**	**Sequence**
circAnks1a site-1	5’-CCUAGGGAACGCCCGCCACCUCCAGCAAAGCCGCCGCCUGAUGAAGAG-3’
circAnks1a site-1 mutant	5’-CCUAGGGAACGCCCGCCACCAGGUCGUUAGCCGCCGCCUGAUGAAGAG-3’
circAnks1a site-2	5’-UGGCUGCAGGAAUCGACGUCAACAUCAAGGACAACCGUGGCCUGACU-3’
circAnks1a site-2 mutant	5’-UGGCUGCAGGAAUCGACGUCUUGUAGUAGGACAACCGUGGCCUGACU-3’

The nucleotide sequences of probes used in Northern blotting (Table 8b)

**Probe Name**	**Sequence**
circAnks1a	5’-UGUCGUUGUUCUUCAGUCCU-3’
mAnks1a	5’-UAUAAGGGUCAAAGGGGCCGUACUUUUUGAGUUCUUUGAUC-3’
