# Supplementary material for: CircAnks1a in the spinal cord regulates hypersensitivity in a rodent model of neuropathic pain
Source: Nat Commun. 2019 Sep 11;10:4119. doi: 10.1038/s41467-019-12049-0 (PMC6739334; doi:10.1038/s41467-019-12049-0)
Supplement: Supplementary file 3 — Reporting Summary [file 41467_2019_12049_MOESM3_ESM.pdf]

nature research

Corresponding author(s): Wen-Jun Xin

Last updated by author(s): Aug 2, 2019

Reporting Summary

Nature Research wishes to improve the reproducibility of the work that we publish. This form provides structure for consistency and transparency in reporting. For further information on Nature Research policies, see Authors & Referees and the Editorial Policy Checklist.

Statistics

For all statistical analyses, confirm that the following items are present in the figure legend, table legend, main text, or Methods section.

n/a	Confirmed	
☐	☒	The exact sample size (*n*) for each experimental group/condition, given as a discrete number and unit of measurement
☐	☒	A statement on whether measurements were taken from distinct samples or whether the same sample was measured repeatedly
☐	☒	The statistical test(s) used AND whether they are one- or two-sided<br>*Only common tests should be described solely by name; describe more complex techniques in the Methods section.*
☒	☐	A description of all covariates tested
☐	☒	A description of any assumptions or corrections, such as tests of normality and adjustment for multiple comparisons
☐	☒	A full description of the statistical parameters including central tendency (e.g. means) or other basic estimates (e.g. regression coefficient) AND variation (e.g. standard deviation) or associated estimates of uncertainty (e.g. confidence intervals)
☐	☒	For null hypothesis testing, the test statistic (e.g. *F*, *t*, *r*) with confidence intervals, effect sizes, degrees of freedom and *P* value noted<br>*Give P values as exact values whenever suitable.*
☒	☐	For Bayesian analysis, information on the choice of priors and Markov chain Monte Carlo settings
☒	☐	For hierarchical and complex designs, identification of the appropriate level for tests and full reporting of outcomes
☒	☐	Estimates of effect sizes (e.g. Cohen's *d*, Pearson's *r*), indicating how they were calculated

Our web collection on statistics for biologists contains articles on many of the points above.

Software and code

Policy information about availability of computer code

Data collection	The RNA-seq fastq reads were mapped to the rat reference genome (Rnor_6.0) using TopHat2. The unmapped reads were then remapped using Tophat-fusion module to identify circRNAs.<br>The whole genome expression microarray data were extracted using Agilent Feature Extraction Software.
Data analysis	HTseq was used to calculate the count values and Reads Per Million mapped reads (RPM) values of circRNAs in each sample and subjected the candidate circRNAs to annotation and length analysis. Differential expression analysis of the RNA-seq was performed using DESeq in R with the default parameters.<br>Differentially expressed genes (DEGs) of microarray data were screened using SAM v4.01 software.

For manuscripts utilizing custom algorithms or software that are central to the research but not yet described in published literature, software must be made available to editors/reviewers. We strongly encourage code deposition in a community repository (e.g. GitHub). See the Nature Research guidelines for submitting code & software for further information.

Data

Policy information about availability of data

All manuscripts must include a data availability statement. This statement should provide the following information, where applicable:

- Accession codes, unique identifiers, or web links for publicly available datasets
- A list of figures that have associated raw data
- A description of any restrictions on data availability

The data for RNA-Seq have been deposited in the SRA database, with the accession code PRJNA558403. All the figures listed in the manuscript have associated raw data. No restrictions on data availability.

Field-specific reporting

Please select the one below that is the best fit for your research. If you are not sure, read the appropriate sections before making your selection.

☒ Life sciences ☐ Behavioural & social sciences ☐ Ecological, evolutionary & environmental sciences

For a reference copy of the document with all sections, see nature.com/documents/nr-reporting-summary-flat.pdf

Life sciences study design

All studies must disclose on these points even when the disclosure is negative.

Sample size	The sample size was determined according to peers' and our previous publications in behavioral and pertinent molecular studies.
Data exclusions	No data were excluded from the analyses.
Replication	All attempts at replication were successful.
Randomization	The samples were randomized using a random number table generated by computer.
Blinding	The investigators were blind to the group allocation during data collection and analysis.

Reporting for specific materials, systems and methods

We require information from authors about some types of materials, experimental systems and methods used in many studies. Here, indicate whether each material, system or method listed is relevant to your study. If you are not sure if a list item applies to your research, read the appropriate section before selecting a response.

Materials & experimental systems

n/a	Involved in the study	
☐	☒	Antibodies
☐	☒	Eukaryotic cell lines
☒	☐	Palaeontology
☐	☒	Animals and other organisms
☒	☐	Human research participants
☒	☐	Clinical data

Methods

n/a	Involved in the study	
☒	☐	ChIP-seq
☒	☐	Flow cytometry
☒	☐	MRI-based neuroimaging

Antibodies

Antibodies used

All antibodies used in the study are listed below.
Anti-VEGFB, Santa, sc-80442
Anti-YBX1, Abcam, ab12148; Biorbyt, orb8480
Anti-transportin-1, GeneTex, GTX103003
Anti-Ago2, Abcam, ab32381
Anti-histone H3, Biorbyt, orb136531
Anti-β-actin, CST, 4967
Anti-GFAP, CST, 3670
Anti-Iba1, Abcam, ab5076
Anti-NeuN, Millipore, MAB377
Cy3 donkey anti-rabbit, Jackson, 711-165-152
Cy3 donkey anti-mouse, Jackson, 715-165-150
Cy3 donkey anti-goat, Jackson, 705-165-003
FITC donkey anti-rabbit, Jackson, 711-095-152
FITC donkey anti-mouse, Jackson, 715-095-150
FITC donkey anti-goat, Jackson, 705-095-003

Validation

The validation of primary antibodies are listed below. WB: Western blotting; IP: immunoprecipitation; IHC: immunohistochemistry; RIP: RNA immunoprecipitation; ChIP: Chromatin immunoprecipitation.
Anti-VEGFB, WB/IHC, rat/mouse
Anti-YBX1, WB/IHC/RIP/ChIP, rat
Anti-transportin-1, IP, rat
Anti-Ago2, WB/RIP, rat
Anti-histone H3, WB, rat
Anti-β-actin, WB, rat/mouse
Anti-GFAP, IHC, rat

	Anti-Iba1, IHC, rat<br>Anti-NeuN, IHC, rat

Eukaryotic cell lines

Policy information about cell lines

Cell line source(s)	The C6 cell line was derived from glial cells of Rats.<br>The HEK 293T cell line was derived from embryonic kidney of human.
Authentication	STR analysis were chosen to perform the authentication.
Mycoplasma contamination	All cell lines tested negative for mycoplasma contamination.
Commonly misidentified lines<br>(See ICLAC register)	None

Animals and other organisms

Policy information about studies involving animals; ARRIVE guidelines recommended for reporting animal research

Laboratory animals	Laboratory animals used in the study are Male Sprague-Dawley rats, weighing 200 to 220 g; mice with C57BL/6 background weighing 20-30 g; and VEGFBflox/flox mice with C57BL/6 background weighing 20-30 g.
Wild animals	None of wild animals involved in the study.
Field-collected samples	The behavioral test was performed at 24℃ temperature and 50% to 60% humidity. After the whole procedure, animals were executed.
Ethics oversight	All experimental procedures were approved by the Local Animal Care Committee and were carried out in accordance with the guidelines of the National Institutes of Health (NIH) on animal care and the ethical guidelines.

Note that full information on the approval of the study protocol must also be provided in the manuscript.
